# Supplementary material for: Studies on Flavor Compounds and Free Amino Acid Dynamic Characteristics of Fermented Pork Loin Ham with a Complex Starter
Source: Foods. 2022 May 21;11(10):1501. doi: 10.3390/foods11101501 (PMC9142104; doi:10.3390/foods11101501)
Supplement: Supplementary file 1 [file foods-11-01501-s001.zip › Figure S1.pdf]

Figure S1. Correlation coefficient plot of VOCs and FAA in Fermented pork loin ham.

A: Correlation coefficient plot of alcohols and FAA

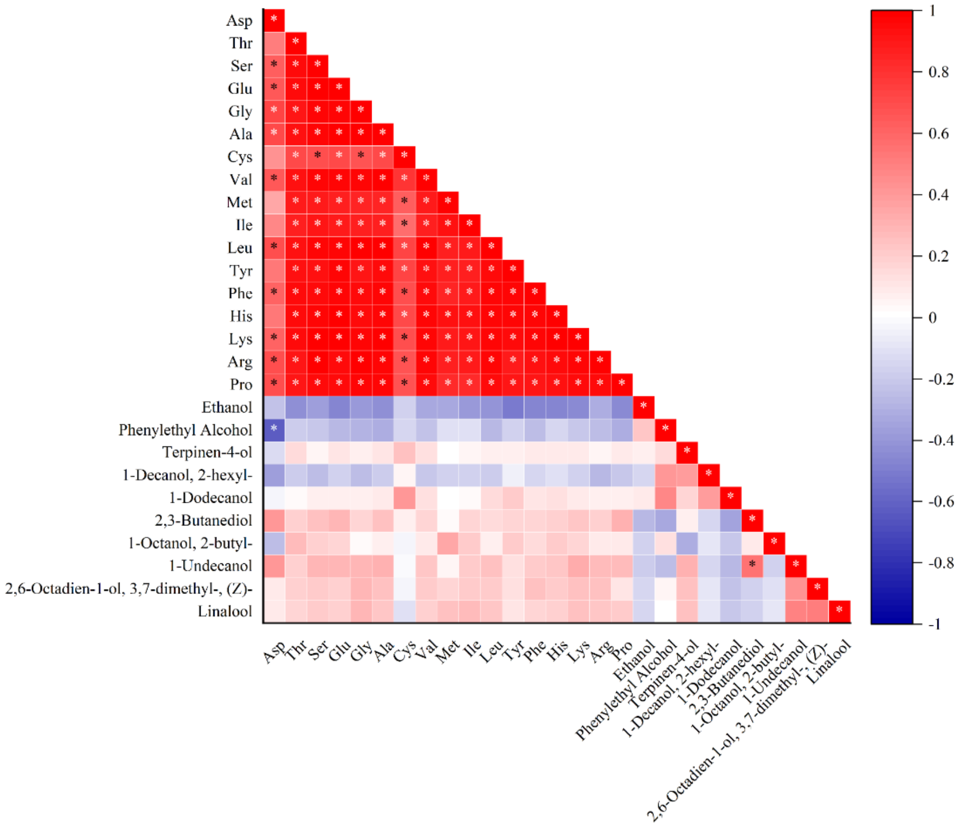

* p<=0.01

B: Correlation coefficient plot of aldehydes and FAA

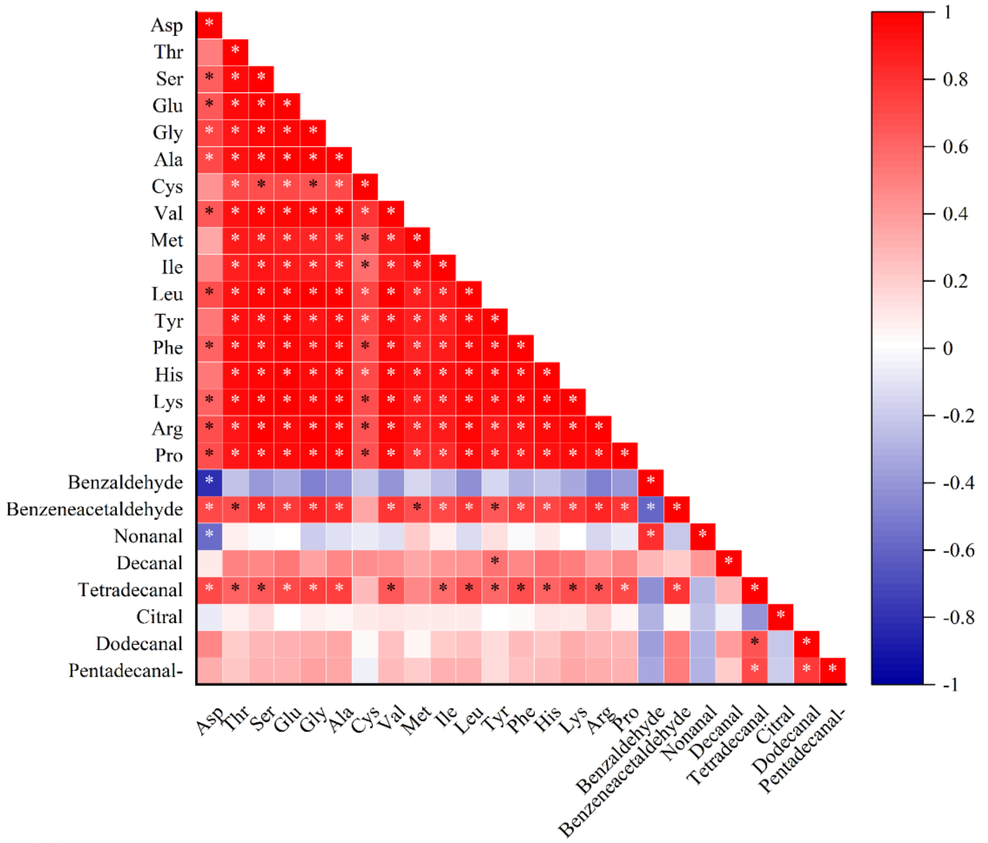

C: Correlation coefficient plot of acids and FAA

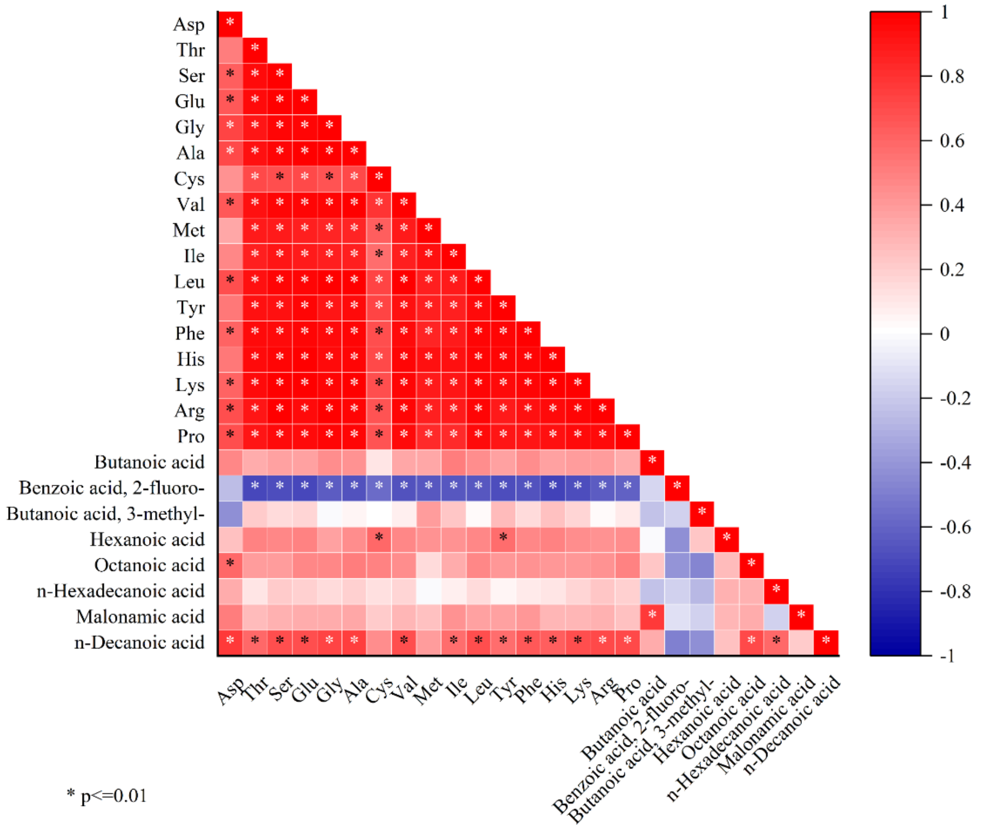

D: Correlation coefficient plot of ketones and FAA

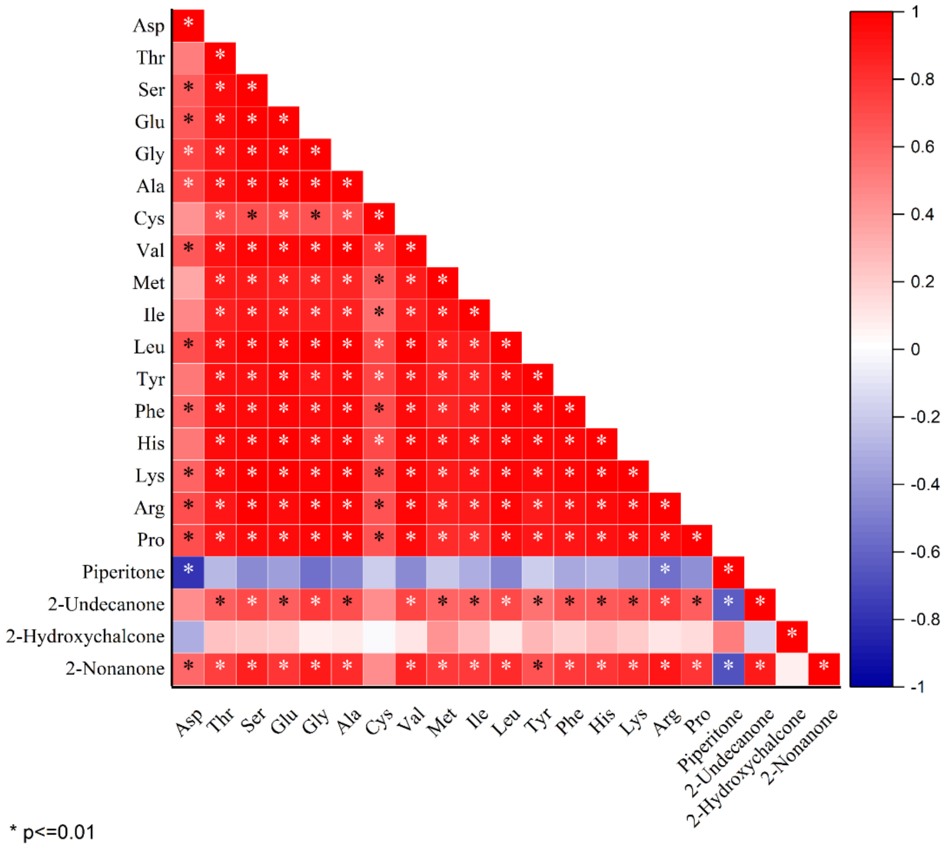

* p<=0.01

E: Correlation coefficient plot of Esters and FAA. *: *p*≤0.01

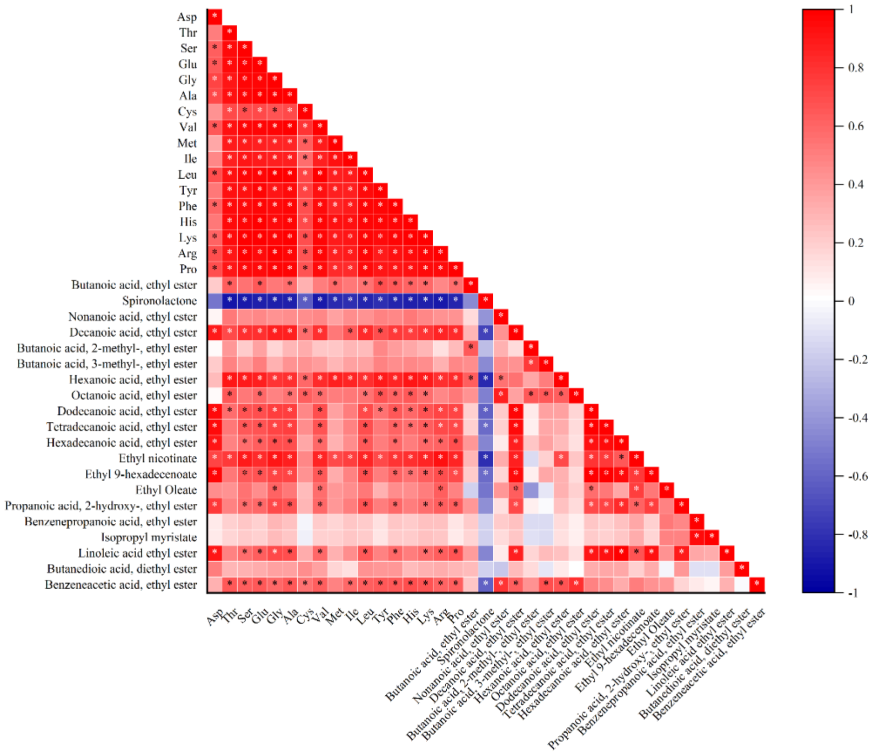

* p<=0.01
