# Supplementary material for: Studies on Flavor Compounds and Free Amino Acid Dynamic Characteristics of Fermented Pork Loin Ham with a Complex Starter
Source: Foods. 2022 May 21;11(10):1501. doi: 10.3390/foods11101501 (PMC9142104; doi:10.3390/foods11101501)
Supplement: Supplementary file 1 [file foods-11-01501-s001.zip › Table S1.pdf]

Table S1 The details of volatile compounds identified by GC-MS

	Number	RT	Compound Name [a]	Cas #	RSI	Fermentation days [b]							Odor type [c]
						0	7	14	21	28	35	42	
Acids	1	6.29	Butanoic acid	107-92-6	803	#N/A	#N/A	#N/A	#N/A	0.06±0.04	0.08±0.01	#N/A	Butter, Cheese, Sour
	2	3.72	Benzoic acid, 2-fluoro-	445-29-4	971	0.1±0.01	#N/A	#N/A	#N/A	#N/A	#N/A	#N/A	N/A
	3	8.31	Isovaleric acid	503-74-2	852	#N/A	0.01±0.01	0.05±0	#N/A	#N/A	#N/A	#N/A	Cheese, Pungent
	4	13.37	Hexanoic acid	142-62-1	863	#N/A	0.1±0.01	0.12±0.01	0.14±0.04	#N/A	0.15±0.01	0.17±0.01	Cheese, Oil, Pungent, Sour
	5	19.67	Octanoic acid	124-07-2	874	#N/A	0.77±0.26	#N/A	0.56±0.06	0.56±0.13	0.96±0.61	1±0.11	Cheese, Fat, Grass, Oil
	6	41.32	n-Hexadecanoic acid	57-10-3	851	#N/A	#N/A	#N/A	0.02±0.01	#N/A	#N/A	0.02±0	Flavoring Agents
	7	25.07	n-Decanoic acid	334-48-5	839	#N/A	0.21±0.03	0.13±0.04	0.41±0.12	0.47±0.07	0.42±0.07	0.63±0.12	Cheese, Pungent
Esters	1	6.79	Butanoic acid, ethyl ester	105-54-4	839	0.04±0.01	0.18±0.02	0.29±0.01	#N/A	0.23±0.03	0.23±0.03	0.16±0.01	Apple, Butter, Cheese, Pineapple, Strawberry
	2	21.45	Spironolactone	52-01-7	832	0.07±0.01	0.05±0.03	#N/A	#N/A	#N/A	#N/A	#N/A	N/A
	3	23.29	Nonanoic acid, ethyl ester	123-29-5	870	0.07±0	0.14±0.01	0.11±0.02	0.13±0.02	0.12±0.02	0.11±0.03	0.11±0.01	Floral
	4	25.97	Decanoic acid, ethyl ester	110-38-3	917	0.26±0.04	2.38±0.26	2.96±0.31	5.11±0.49	6.65±1	7.57±0.78	8.39±0.59	Brandy, Grape, Pear

Number	RT	Compound Name [a]	Cas #	RSI	Fermentation days [b] 0	7	14	21	28	35	42	Odor type [c]
5	8.57	Butanoic acid, 2-methyl-, ethyl ester	7452-79-1	937	#N/A	0.12±0.03	0.07±0	#N/A	0.04±0	0.07±0.03	0.08±0.01	Apple, Ester, Green Apple, Kiwi, Strawberry
6	8.76	Butanoic acid, 3-methyl-, ethyl ester	108-64-5	852	#N/A	0.99±0.1	0.74±0.04	0.44±0.05	0.47±0.01	0.5±0.03	1.24±0.1	Apple, Fruit, Pineapple, Sour
7	14.08	Hexanoic acid, ethyl ester	123-66-0	915	#N/A	1.07±0.06	1.99±0.41	1.43±0.1	1.5±0.03	1.41±0.02	1.38±0.03	Apple Peel, Brandy, Fruit Gum, Overripe Fruit, Pineapple
8	20.43	Octanoic acid, ethyl ester	106-32-1	947	#N/A	24.3±1.67	14.47±1.04	15.84±2.39	15.73±1.08	16±2.17	14.09±0.59	Apricot, Brandy, Fat, Floral, Pineapple
9	32.02	Dodecanoic acid, ethyl ester	106-33-2	821	#N/A	0.02±0	0.05±0.01	0.19±0.02	0.28±0.05	0.36±0.01	0.45±0.04	Floral, Fruit, Leaf
10	37.59	Tetradecanoic acid, ethyl ester	124-06-1	807	#N/A	0.01±0	0.05±0.01	0.27±0.02	0.45±0.04	0.61±0.07	0.74±0.02	Wax
11	42.06	Hexadecanoic acid, ethyl ester	628-97-7	852	#N/A	0.06±0.01	0.19±0.03	1.1±0.05	1.78±0.09	2.29±0.25	2.57±0.01	Wax
12	20.93	Ethyl nicotinate	614-18-6	855	#N/A	#N/A	0.1±0.01	0.13±0.02	0.15±0.01	0.13±0.01	0.14±0.01	Flavoring Agents
13	41.60	Ethyl 9-hexadecenoate	54546-22-4	800	#N/A	#N/A	0.02±0	0.11±0	0.2±0.01	0.27±0.04	0.32±0.01	Dairy
14	45.43	Ethyl Oleate	111-62-6	886	#N/A	#N/A	#N/A	0.27±0.05	0.3±0.03	0.11±0.01	0.13±0	Dairy

	Number	RT	Compound Name [a]	Cas #	RSI	Fermentation days [b] 0	7	14	21	28	35	42	Odor type [c]
	15	7.20	Propanoic acid, 2-hydroxy-, ethyl ester	97-64-3	909	#N/A	#N/A	#N/A	#N/A	0.9±0.18	0.91±0.16	0.41±0.15	Cheese, Floral, Fruit, Pungent, Rubber
	16	24.73	Benzenepropanoic acid, ethyl ester	2021-28-5	810	#N/A	#N/A	#N/A	#N/A	0.04±0	#N/A	#N/A	Flower, Honey
	17	38.35	Isopropyl myristate	110-27-0	859	#N/A	#N/A	#N/A	#N/A	0.04±0.02	#N/A	#N/A	Dairy
	18	45.30	Linoleic acid ethyl ester	544-35-4	865	#N/A	#N/A	#N/A	#N/A	0.09±0.01	0.1±0.01	0.1±0	Flavoring Agents
	19	19.91	Butanedioic acid, diethyl ester	123-25-1	818	#N/A	#N/A	#N/A	#N/A	#N/A	0.01±0	#N/A	Cotton, Fabric, Floral, Fruit, Wine
	20	21.75	Benzeneacetic acid, ethyl ester	101-97-3	868	#N/A	0.04±0.02	0.03±0.01	0.04±0.01	0.04±0	0.03±0	0.05±0	Floral, Fruit, Honey, Rose
Alcohols	1	20.58	Ethanol	64-17-5	890	0.18±0.01	#N/A	#N/A	0.35±0.01	0.01±0	#N/A	#N/A	Flavoring Agents
	2	17.82	Phenylethyl Alcohol	60-12-8	908	0.14±0.01	0.19±0.08	0.16±0.08	0.19±0.03	0.14±0.02	0.09±0.01	0.07±0.01	Fruit, Honey, Lilac, Rose, Wine
	3	19.97	Terpinen-4-ol	562-74-3	860	#N/A	0.06±0	#N/A	0.05±0	0.04±0	#N/A	0.04±0	Earth, Must, Nutmeg, Wood
	4	23.88	1-Decanol, 2-hexyl-	2425-77-6	868	#N/A	0.03±0.03	#N/A	#N/A	#N/A	#N/A	#N/A	N/A

	Number	RT	Compound Name [a]	Cas #	RSI	Fermentation days [b] 0	7	14	21	28	35	42	Odor type [c]
	5	28.07	1-Dodecanol	112-53-8	920	#N/A	0.04±0.03	#N/A	0.03±0.01	#N/A	0.03±0.01	#N/A	Flavoring Agents
	6	6.54	2,3-Butanediol	513-85-9	938	#N/A	#N/A	0.06±0.01	#N/A	#N/A	#N/A	0.14±0.01	Flavoring Agents
	7	19.32	1-Octanol, 2-butyl-	3913-02-8	832	#N/A	#N/A	0.04±0.02	#N/A	#N/A	#N/A	#N/A	N/A
	8	28.09	1-Undecanol	112-42-5	926	#N/A	#N/A	#N/A	0.02±0	0.07±0.06	#N/A	0.07±0.03	Mandarin
	9	22.00	2,6-Octadien-1-ol, 3,7-dimethyl-, (Z)-	106-25-2	830	#N/A	#N/A	#N/A	#N/A	0.03±0	#N/A	#N/A	Floral, Fruit
	10	17.47	Linalool	78-70-6	844	#N/A	#N/A	#N/A	#N/A	0.32±0.13	#N/A	#N/A	N/A
Aldehydes	1	12.74	Benzaldehyde	100-52-7	895	0.28±0.02	0.53±0.07	0.48±0.07	0.13±0.01	0.18±0.02	0.09±0.01	0.11±0.02	Bitter Almond, Burnt Sugar, Cherry, Malt, Roasted Pepper
	2	15.60	Benzeneacetaldehyde	122-78-1	887	0.05±0.01	#N/A	0.22±0.01	0.19±0.01	0.32±0.04	0.23±0.04	0.26±0.01	Berry, Geranium, Honey, Nut, Pungent
	3	17.63	Nonanal	124-19-6	924	0.89±0.01	1.59±0.35	3.08±0.27	0.6±0.02	0.56±0.12	0.52±0.1	0.72±0.02	Fat, Floral, Green, Lemon
	4	20.72	Decanal	112-31-2	899	#N/A	0.14±0.07	0.14±0.06	0.09±0.01	0.09±0.05	0.08±0.04	0.15±0.01	Floral, Fried, Orange Peel, Penetrating, Tallow

	Number	RT	Compound Name [a]	Cas #	RSI	Fermentation days [b] 0	7	14	21	28	35	42	Odor type [c]
	5	38.17	Tetradecanal	124-25-4	948	#N/A	0.15±0.01	0.38±0.01	0.02±0.01	1.12±0.11	0.76±0.03	0.83±0.02	Flavoring Agents
	6	22.51	Citral	5392-40-5	943	#N/A	#N/A	#N/A	0.08±0.04	#N/A	#N/A	#N/A	Lemon
	7	26.38	Dodecanal	112-54-9	868	#N/A	#N/A	#N/A	#N/A	0.02±0	#N/A	0.02±0	Citrus, Fat, Lily
	8	35.60	Pentadecanal-	2765-11-9	888	#N/A	#N/A	#N/A	#N/A	0.02±0.01	#N/A	0.01±0	N/A
Ketones	1	22.13	2-Cyclohexen-1-one, 3-methyl-6-(1-methylethyl)-	89-81-6	803	0.04±0.01	0.11±0.01	0.09±0.02	#N/A	#N/A	#N/A	#N/A	Flavoring Agents
	2	22.78	2-Undecanone	112-12-9	830	#N/A	0.03±0.01	0.06±0.01	0.15±0.02	0.16±0.03	0.11±0.01	0.09±0.02	Fresh, Green, Orange, Rose
	3	7.64	2-Hydroxychalcone	644-78-0	963	#N/A	#N/A	0.03±0.01	#N/A	#N/A	#N/A	#N/A	N/A
	4	17.16	2-Nonanone	821-55-6	916	#N/A	#N/A	0.15±0.01	0.22±0.02	0.25±0.02	0.18±0.01	0.15±0.01	Fragrant, Fruit, Green, Hot Milk

a: Identification by GC/MS.

b: Volatile compounds produced (expressed as AU × 10^8) in fermented tenderloin at different times. Values are presented as “MEAN ± SD”. #N/A means not detected

c: Odor descriptions were mainly gathered from online database (https://pubchem.ncbi.nlm.nih.gov/)
